# The SNAP hypothesis: Chromosomal rearrangements could emerge from positive Selection during Niche Adaptation

**DOI:** 10.1371/journal.pgen.1008615

**Published:** 2020-03-04

**Authors:** Gerrit Brandis, Diarmaid Hughes

**Affiliations:** Department of Medical Biochemistry and Microbiology, Biomedical Center, Uppsala University, Uppsala, Sweden; Institut Pasteur, FRANCE

## Abstract

The relative linear order of most genes on bacterial chromosomes is not conserved over evolutionary timescales. One explanation is that selection is weak, allowing recombination to randomize gene order by genetic drift. However, most chromosomal rearrangements are deleterious to fitness. In contrast, we propose the hypothesis that rearrangements in gene order are more likely the result of selection during niche adaptation (SNAP). Partial chromosomal duplications occur very frequently by recombination between direct repeat sequences. Duplicated regions may contain tens to hundreds of genes and segregate quickly unless maintained by selection. Bacteria exposed to non-lethal selections (for example, a requirement to grow on a poor nutrient) can adapt by maintaining a duplication that includes a gene that improves relative fitness. Further improvements in fitness result from the loss or inactivation of non-selected genes within each copy of the duplication. When genes that are essential in single copy are lost from different copies of the duplication, segregation is prevented even if the original selection is lifted. Functional gene loss continues until a new genetic equilibrium is reached. The outcome is a rearranged gene order. Mathematical modelling shows that this process of positive selection to adapt to a new niche can rapidly drive rearrangements in gene order to fixation. Signature features (duplication formation and divergence) of the SNAP model were identified in natural isolates from multiple species showing that the initial two steps in the SNAP process can occur with a remarkably high frequency. Further bioinformatic and experimental analyses are required to test if and to which extend the SNAP process acts on bacterial genomes.

## Author summary

All life on earth has evolved from a universal common ancestor with a specific order of genes on the chromosome. This order is not maintained in modern species and the standard hypothesis is that changes reflect a lack of strong selection on gene order. Here, we propose an alternative hypothesis, SNAP. The occupation of a novel environment by bacteria is generally a trade-off situation. For example, while the bacteria may not be adapted to grow well under the new conditions, they may benefit by not having to share available resources with other microorganisms. Bacterial populations frequently acquire duplications of chromosomal segments containing genes that can help them adapt to a new environment. Other genes that are also duplicated are not required in two copies so that over time a superfluous copy can be lost. Eventually, the process of duplication and gene loss can lead to the rearrangement of the gene order in the chromosomal segment. The major benefit of this model over the standard hypothesis is that the process is driven by positive selection and can reach fixation rapidly.

## Introduction

Genetic information is encoded in nucleic acid chromosomes organized as linear sequences of genes. Comparative genomic analyses support the hypothesis that life on earth has evolved from a universal common ancestor [1–6]. This genetic diversity of life reflects the interplay between selection for organisms to occupy and thrive in different environmental niches, and the operation of mechanisms that can change the existing nucleic acid sequence in a chromosome. The mechanisms of genetic change are errors in the accuracy of chromosome replication, and the recombination of sequences within and between chromosomes. The former mechanism can lead to sequence divergence between homologous genes in separate species, whereas the latter mechanism can create novel genes by fusion or splitting of existing genes, and can also move genes from one chromosomal location to another. Because organisms must maintain a high level of relative fitness to compete for resources to support survival, growth and replication, changes in individual gene sequences are often subject to selection to maintain or adapt their functionality in particular environments.

The relationship between selection, conservation of gene order on chromosomes, and relative bacterial fitness in different environments is less obvious. The requirement to integrate gene expression with chromosome replication is one force that shapes the linear organization of bacterial chromosomes. Bacterial genes are most often co-oriented with the direction of replication, and most of the highly conserved and highly expressed genes are located in the half of the chromosome closest to the origin of replication [7]. This replication-related selection can minimise transcription-translation collisions and takes advantage of gene dosage effects to increase expression of some genes but it is not clear that it explicitly selects for maintenance of an ancient linear gene order. A remarkable example of conservation of an ancient co-linear organization of gene is found for a large set of genes involved in transcription and translation [8]. This conserved cluster of operons includes: *S10* (11 ribosomal proteins), *spc* (11 ribosomal proteins and SecY), *alpha* (4 ribosomal proteins and RpoA), *rrnB* (3 ribosomal rRNA and 2 tRNA genes), *tufB* (4 tRNA genes, EF-TuB), *secE* (SecE, NusG), *rpoBC* (4 ribosomal proteins, RpoB and RpoC) and *str* (2 ribosomal proteins, EF-G and EF-TuA). This gene/operon cluster was present in the last common ancestor of the bacteria and archaea [9–11]. Although in many species some of these operons have become separated by gene insertions, the ancient organization is conserved in many of the *Enterobacteriaceae* [12–15]. The underlying selective mechanism has recently been linked to these operons being concatenated [16]. By experimentally manipulating one of the contiguous operon pairs, *tufB*-*secE* in *Salmonella*, it was shown that an inter-operon terminator-promoter overlap has a significant role in regulating gene expression and its interruption significantly reduces bacterial fitness. The other operons of the ancient cluster that remain contiguous in *Salmonella* (*tufB*-*secE*-*rpoBC* and *S10*-*spc*-*alpha*) are also each connected by an inter-operon terminator-promoter overlap. Accordingly, it was proposed that the concatenation of operons is an ancient feature of some operons that restricts the potential to rearrange particular regions of bacterial chromosomes and selects for the maintenance of a co-linear operon organization over billions of years [16].

The organization of many bacterial genes into multigene transcriptional units, operons, also suggests mechanisms that could act to conserve linear gene order [17–19]. Within operons gene order might be maintained by selection for co-regulation, or for horizontal transfer of a fully functional unit. However, even the conservation of operon organization is generally low over evolutionary time spans for distantly related species [20, 21], although there are exceptions, for example, *E*. *coli* and *S*. *enterica*, where despite greater than 100 Myr of separation, co-linear gene order within operons, and throughout the chromosome is remarkably conserved [15, 22].

In spite of the examples of conservation above, the linear organization of homologous genes on bacterial chromosomes of different species is highly variable and for most homologous genes there is no long-range co-linearity in gene order [23, 24]. The standard interpretation for the low level of conservation is that selection to maintain linear gene order is weak and this allows changes in gene order to occur by genetic drift. In contrast, an *in silico* study of contiguous gene pairs across 126 bacterial genomes of different species found that the maintenance of contiguity was actually higher than predicted by experimental parameters, even for gene pairs not in operons, suggesting that many gene order rearrangements are deleterious and that purifying selection is operating [25]. This paradox could be resolved if gene order rearrangements during speciation did not arise primarily by genetic drift but were instead selected. We propose a radical alternative to the drift hypothesis: Selection for Niche Adaptation. The SNAP hypothesis, proposes that changes in relative gene order on bacterial chromosomes are driven by selection. During evolution the organisms that succeed are those that can best adapt to the available environmental niches (survival of the fittest). Such niches are not constant but can arise or change over time as a result of changes in environmental conditions, and because of changes wrought by the interactions of different organisms with both the organic and the physical environments. Our hypothesis is that rearrangements in chromosomal gene order can be selected indirectly as a result of selection acting on organisms (in particular microorganisms) to adapt to changing or novel environmental niches. On an evolutionary timescale the chromosomes of organisms adapting to a new niche would very rapidly ‘snap’ into a new gene order organization. The SNAP hypothesis is explained in words and figures in the text below, and modelled mathematically using reasonable experimentally-derived parameters.

## Results

### Genetic drift hypothesis

**Fig 1:**
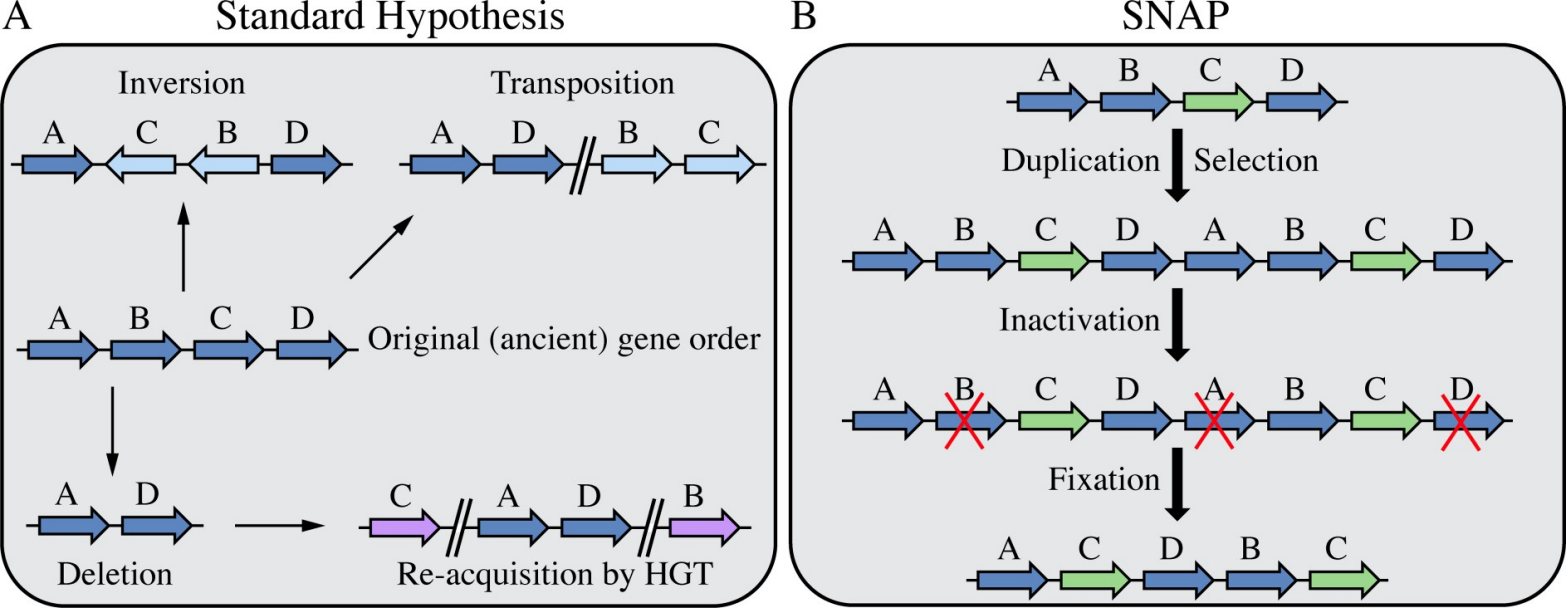
Comparison of standard hypothesis of genomic rearrangements and the SNAP model. (**A**) Changes in gene order caused by inversions, transpositions, deletions and re-acquisition. Genes in their original location are shown in dark blue, novel locations are indicated in light blue and genes acquired by horizontal gene transfer are shown in purple. (**B**) Selection under niche adaptation (SNAP). The gene under selection for duplication is shown in green, genes inactivated are marked with a red X.

In the standard model, gene order on chromosomes is assumed to be under very weak selection and therefore subject to evolution by genetic drift associated with recombination. Several different types of recombinational event could be involved in rearranging the order of genes on a chromosome: inversion, transposition, deletion, and the acquisition of homologous genes by horizontal gene transfer (Fig 1A). In principle, the successive occurrence of one or more of these types of recombination event could ultimately lead to a significant rearrangement in the linear order of genes on a chromosome. However, in practice the relative fitness of intermediates, and the rates associated with each step in the process, will impose severe limitations on the drift hypothesis as a primary explanation for gene order rearrangements. For an environmentally well-adapted organism there will, in most cases, be no selective benefit associated with inverting, deleting, or transposing a chromosomal segment. Similarly, acquiring additional copies of existing genes by HGT and their insertion at a novel location is unlikely to increase fitness. Deletion or impairment of any essential gene will be lethal or will severely reduce fitness. For most non-lethal chromosomal rearrangements the expectation is that at best they will be neutral but are more likely to have a negative effect on relative fitness [25]. It is unlikely that chromosomal rearrangements, even when they are neutral with respect to fitness, will increase in frequency and reach fixation in a population. A second limitation on the drift hypothesis is the low frequency with which individual non-lethal recombination events, such as inversions, occur in bacterial populations [26–29]. Significant gene order rearrangements between species would require a succession of non-lethal recombination events, each occurring with a low probability, and each reaching fixation in a population, to generate a significant shuffling of gene order as observed when comparing different species [23–25]. In summary, while the recombinational mechanisms illustrated in Fig 1A could promote genome fluidity over successive cycles, if each event occurs at a low frequency, and without a positive selection, fixation would depend strongly on founder effects (small population bottlenecks). We do not rule out genetic drift as a contributing factor in gene order rearrangements but we think that our alternative hypothesis, SNAP, has some significant advantages in terms of the probability of occurring and being selected to fixation.

### The SNAP hypothesis

SNAP, Selection during Niche Adaptation is based on a sequential series of high frequency events and is driven by selection to fixation (Fig 1B). The SNAP hypothesis involves four sequential stages: **Duplication, Selection, Inactivation, and Fixation**.

**(i) Duplication.** Duplication of segments of a bacterial chromosome is a very frequent event, occurring spontaneously at rates of >10^−2^ to 10^−5^ by recombination between repetitive sequences [30]. The regions duplicated can vary in size from tens of kilobases up to more than a megabase [30–32]. Duplications are intrinsically unstable and segregate unless maintained by selection [33].

**(ii) Selection.** Bacteria frequently live in sub-optimal environments, for example habitats that are nutrient-poor or mildly toxic. Under such conditions, duplications will be selectively maintained if they confer a fitness advantage, for example, if increased dosage of a nutrient transporter gene improves relative fitness [32]. Exposure to antibiotics is also known to select duplications, for example when the bacteria carry a gene encoding a sub-optimal antibiotic-degrading enzyme [34, 35]. In such cases the increase in gene dosage associated with a duplication or amplification provides a strong selective benefit in the particular environmental niche. In addition to having a gene dosage effect, a duplication could also confer a selective advantage by placing a gene under the control of an alternative potent promoter thus increasing its expression or altering its regulation [36]. Adaptive duplications could also be selected for fast growth in nutrient-rich environments. An example could be the occurrence of multiple *rrn* operons in many microbial species that may be a selected genetic mechanism contributing to fast growth [37–41]. Also, the frequently observed duplication of the *tuf* gene, encoding elongation factor EF-Tu, may have been selected in different bacterial species because this duplication helps support faster growth rates than are supported by a single gene copy [42–44].

**(iii) Inactivation.** A duplication is a double-edged sword. The regional duplication will be maintained by selection on the relevant gene(s) but the other genes in the duplicated region will not be under positive selection. Accordingly, most duplicated genes, even those that are essential as single copy genes, can accumulate mutations, either because they are not essential as duplicates, or because their duplication reduces fitness (resource wastage, interference with normal physiology) and there is a positive selection to remove their activity [45]. This process inevitably leads to the accumulation of inactivating mutations in the genes of the duplicated region that are not under positive selection. Gene-inactivating mutations (for example, frameshift, nonsense, deletion) occur with spontaneous rates of 10^−5^ to 10^−6^ per gene [46, 47]. Recombination between repeat sequences that lie within the duplicated region (IS elements for example, or other repeat sequences) could lead to a loss of parts of a duplication, including a copy of an essential gene, at much higher rates. We make the reasonable assumption that gene inactivation mutation will occur randomly with respect to each copy of the duplication.

**(iv) Fixation.** Inactivation of a different essential gene (or a gene required for high fitness) in each copy of the duplicated region will prevent segregation of the duplication. At this stage the duplication is fixed and the net outcome is a chromosome in which the remaining active genes have a rearranged order relative to the ancestral order (see Fig 1B). The remaining duplicated genes can continue to accumulate mutations (including deletions) in each copy of the duplicated region contributing further to rearrangements of the original gene order. In *E*. *coli* there are over 350 chromosomal genes that are essential for growth under rich medium conditions [48] but in general bacteria will have many other genes where inactivation would significantly reduce fitness, or be incompatible with growth under a variety of specific conditions [49–51]. Accordingly, a duplicated region of 100 kb is likely to contain several essential genes providing mutational targets where inactivation will result in fixation of the duplication and a rearranged gene order on the chromosome.

The SNAP hypothesis does not rule out a role for genetic drift in causing gene order rearrangements. It is an alternative mechanism that has very significant advantages compared to genetic drift: it is associated with natural selection (bacteria adapting to a new environment), it is initiated at a very high frequency (spontaneous duplications), it is irreversible (once essential genes have been inactivated in each arm of the duplication), and it is driven to fixation by positive selection. Accordingly, we propose that **positive selection** might play a major role in driving change in the relative order of most genes on a bacterial chromosome.

### Mathematical modelling of SNAP

**Fig 2:**
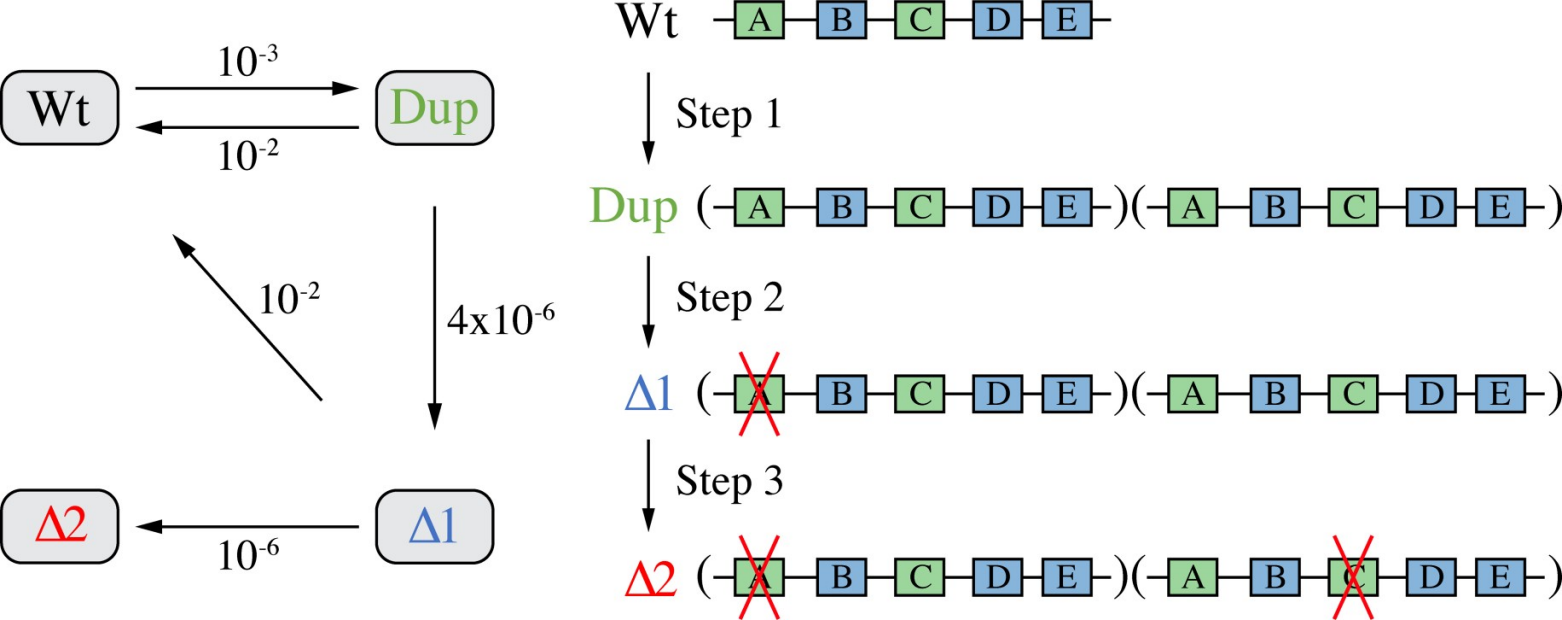
Outline of the minimal population dynamics model. The model allows the appearance of four types of cells: wild-type cells (Wt), cells with a duplication (Dup) of a region that includes two essential genes (green), and cells with the duplication and a single (Δ1) or double (Δ2) inactivation of essential genes. All possible directions and rates of evolution are displayed and the inactivation of two essential genes is assumed to stabilize the duplication.

A minimal mathematical model of SNAP is presented in Fig 2. The spontaneous rates of duplication and mutational gene inactivation used in modelling are taken from published literature [30, 46, 47]. The only variable parameter in the model is the range of potential effects on relative fitness of duplications and mutations within duplicated regions. The model makes the following assumptions: (i) regional duplications occur and can be maintained by selection for a phenotype that is satisfied by duplication of one or more genes encoded within the duplicated region; (ii) the duplicated regions contain at least two essential genes; (iii) gene inactivating mutations occur with normal rates and can inactivate different essential genes in each copy of the duplicated region; (iv) once two different essential genes have been inactivated in different copies of the duplicated region the duplication can no longer segregate to a single copy while maintaining the original gene order.

In this model the wild-type spontaneously generates duplications that are stabilized by a selection for a phenotype (step 1). An essential gene within one copy of the duplicated region is mutationally inactivated (step 2). At this stage there are alternative paths. If the duplication is maintained there is the opportunity for an essential gene within the second copy of the duplicated region to be mutationally inactivated (step 3). Step 3 stabilizes the duplication with a novel linear gene order. Alternatively, if the duplication segregates (for example, because selection is relieved) the original gene order will be maintained. The minimal model is illustrated here with rates for each step that are conservative estimates based on experimentally determined values [30, 46, 47].

**Fig 3:**
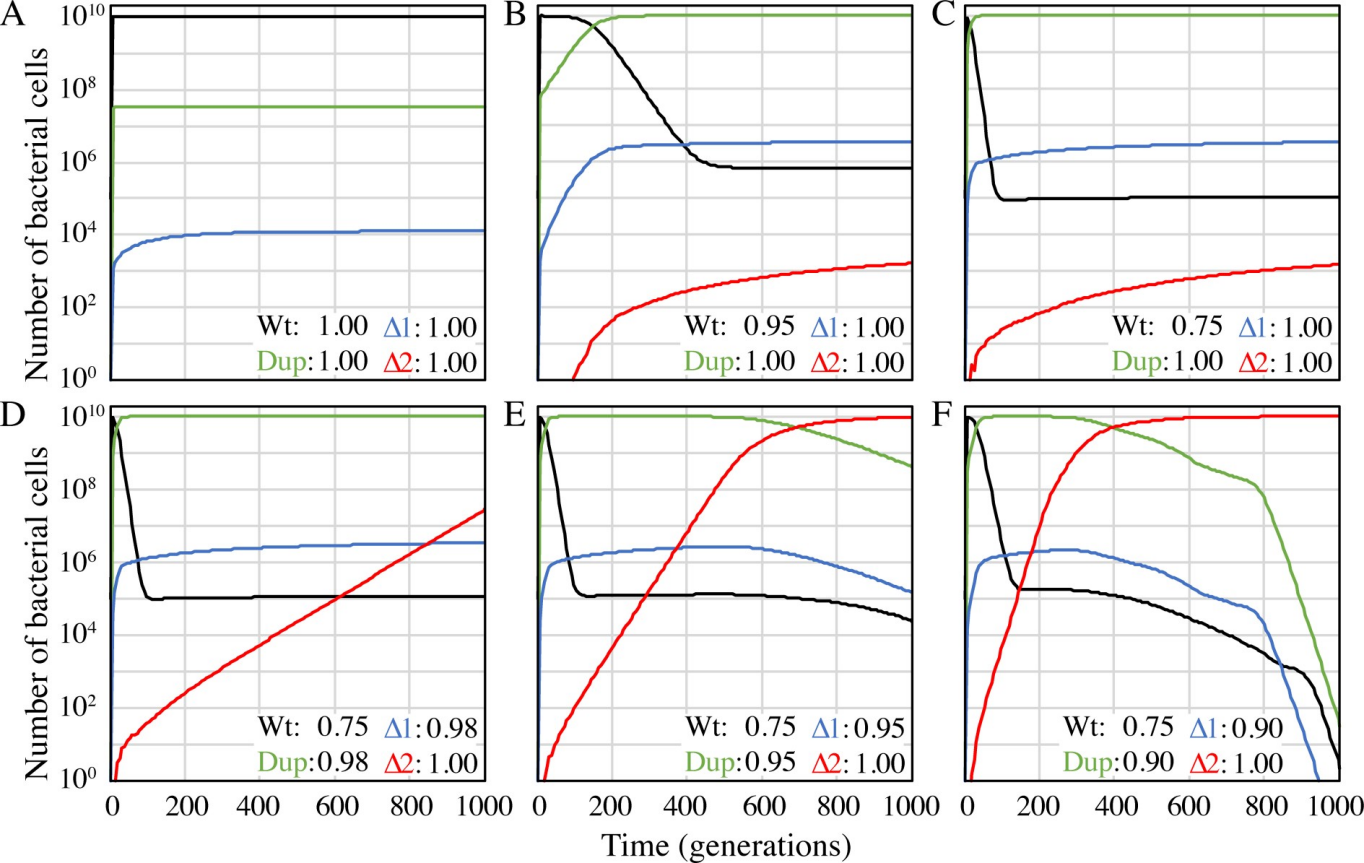
Modelled population dynamics under varying selective conditions. Number of wild-type cells (Wt, black), cells with the duplication (Dup, green) and cells with the duplication that carry a single (Δ1, blue) or double (Δ2, red) essential gene inactivation mutation are shown as a function of time. (**A**-**C**) Strains carrying the duplication have, relative to the wild-type (**A**) equal fitness, (**B**) 5% fitness advantage, or (**C**) 25% fitness advantage. (**D**-**F**) Illustrate panel (**C**) with the added assumption that deleting unnecessary duplicate genes (Δ2) confers a fitness advantage of (**D**) 2%, (**E**) 5%, or (**F**) 10%. All models were run as serial transfers with a starting population of 10^6^ wild-type cells, a total population size of 10^10^ cells and 10^8^ cells transferred per bottleneck. The appearance and reversion of mutant populations was determined by a Monte Carlo procedure based on the frequencies displayed in Fig 2. The fitness parameters for the populations are shown in each panel. All graphs display the average of 100 independent runs. Models were run with Berkeley Madonna (version 9.1.14).

Using this minimal mathematical model, we have measured how changing the values assigned to the fitness parameters would influence the probability of fixing a rearranged gene order (Fig 3). In the absence of any selection or fitness costs, duplication and single gene inactivation occurs and reaches a steady state but does not go to fixation (Fig 3, panel A). Once selection and fitness costs are introduced (a novel environment where the duplication has a fitness advantage over the wild-type) the population carrying duplications increases dramatically and sub-populations carrying the single and double gene inactivation mutations increase in frequency (Fig 3, panels B and C). Adding the assumption that carrying duplicate genes confers a fitness cost leads to the rapid increase and subsequent fixation of the mutant population with the novel gene order (double gene inactivation) (Fig 3, panels D-F). This minimal model suggests that a novel gene order can be generated within a small number of generations if the initial duplication has a selective benefit over the wild-type and the inactivation of duplicate genes from either of the copies further improves fitness.

There are several additional features, that for simplicity, have been omitted from this minimal model but which may play either a restrictive or a positive role in this evolutionary process in different species, or under different selective conditions.

- A feature of the model that potentially restricts its influence on genome rearrangements is the requirement that at least two essential genes be contained within the duplicated region. Essential genes are not expected to be evenly distributed throughout the genome, in which case for some duplications there might never be a transition from step 2 to step 3. This restriction will mostly affect smaller duplications in regions of the chromosome that are poor in essential genes but is less likely to affect large duplications. A counter argument is that under the actual conditions that are selective for maintenance of a duplication (e.g., growth in a challenging niche) many additional genes, even if not essential under all conditions, may be under strong selection to maintain fitness [44, 49, 50].
- A feature of the model that potentially promotes gene order rearrangements is that many duplications will result in unbalanced chromosome replichores. These mutants will be under selection not only to maintain the duplication but also to rebalance their replichores so as to reduce associated fitness costs [52–57]. An improvement in replichore balance could be achieved by a deletion or an inversion. Chromosomes that have undergone a process of duplication followed by inversion will be locked into a structure where the duplication can no longer easily be segregated. This sequence of events can help to promote genome rearrangements by effectively stabilizing a duplication even if the original selection is relieved.
- The evolutionary process does not stop after an essential gene has been inactivated in each duplicated region. The fitness costs associated with having tens to hundreds of genes duplicated will act as a driving force for the continued selection and fixation of mutants that delete or otherwise inactivate all non-required extra copies of duplicated genes where such duplications have a negative impact on fitness.
- Another feature that could promote rapid genetic change is the high prevalence of bacteria that are mutator clones with high mutation rates. Mutator bacteria are estimated to be up to 1% of natural isolates [58–60], and even higher among some clinical isolates [61]. Mutator clones, including those caused by inactivation of the mismatch repair system, have not only a significantly increased rate of point mutation [62] but also a significantly higher rate of recombination that can cause chromosomal rearrangements including duplications, deletions and inversions [27, 63, 64]. Recombinational gene inactivation could also be caused by the movement of IS elements and transposons, the frequency of which will vary between species and potentially be influenced by the environment. With regard to mobile genetic elements (MGE) we note that care must be taken in estimating the number of duplications in genome sequences, to distinguish between those involving non-mobile sequences (the main focus of the SNAP hypothesis) and duplications arising from the movement of MGEs resulting in increased copy number.

Gene inactivation by point mutations occurring at a normal mutation rate (as modelled in Fig 3) leads to a very conservative estimate of gene inactivation rates, and if instead, deletion and insertional inactivation events dominate, and mutators play a significant role, then the rates of gene inactivation within a duplicated region of the chromosome could be much higher than in our simple model.

### Identification of duplications in natural isolates

**Fig 4:**
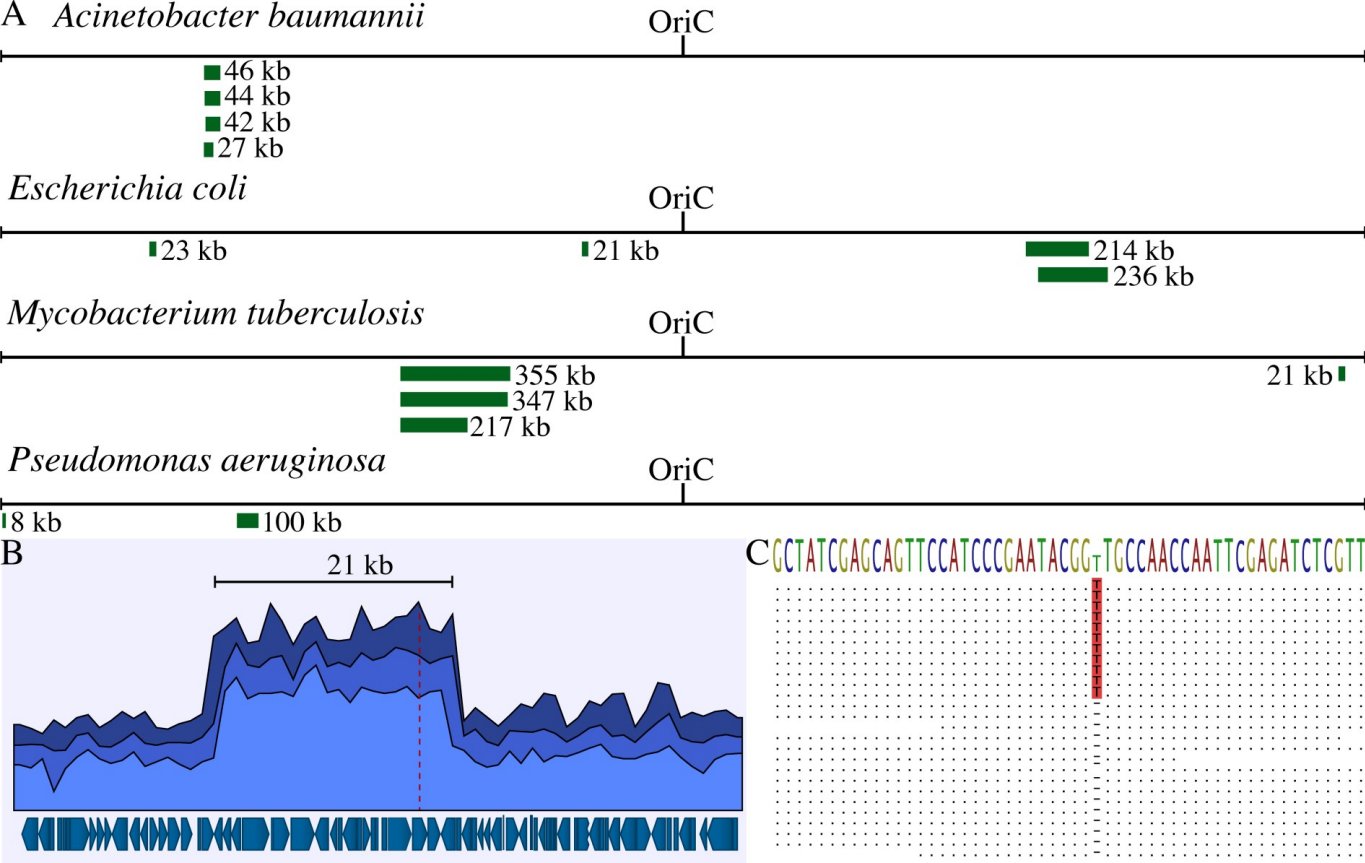
Duplications identified in natural isolates of *A*. *baumannii*, *E*. *coli*, *M*. *tuberculosis*, and *P*. *aeruginosa*. (**A**) One hundred whole genome sequences per species were downloaded from the SRA and analysed for regions with increased coverage. Duplicated regions are indicated with green bars and represent unique segments of the chromosomes. (**B**) Read coverage analysis of a chromosomal section within the *M*. *tuberculosis* isolate with a 21 kb duplication. The blue shades (top to bottom) represent the maximum, average and minimum read coverage on a sliding 1 kb window. Genes within the chromosomal segment are indicated below. The duplicated region contains 21 genes and the frameshift mutation that is present in one copy of the glutamine synthetase gene is indicated with a dotted red line. (**C**) Sequence analysis of the frameshift insertion within the glutamine synthetase gene (~25% of reads shown). The consensus sequence is shown as sequence logo on the top with the reads below. Residues in the reads that match the reference are shown as dots. The insertion of a thymine is indicated in red. The site of the insertion has a 155-fold coverage and the frameshift present in 49% of reads.

Available genome sequences from clinical and environmental isolates of *Acinetobacter baumannii*, *Escherichia coli*, *Mycobacterium tuberculosis*, and *Pseudomonas aeruginosa* were analysed to identify signature features (duplication formation and divergence) of the SNAP model. One hundred genome sequences for each species were downloaded from the Sequence Read Archive (SRA), assembled to a respective standard reference sequence, and duplications were identified based on increased sequence coverage. Duplications were present in 2–4% of the isolates of each species and ranged in length from 8 to 355 kb (Fig 4A). Further analysis of the duplicated sequences showed that two of the fourteen isolates (14%) contained diverging duplications, identified as having a mutation present in ~50% of the reads: A *M*. *tuberculosis* isolate had a frameshift mutation in one copy of MRA_RS09940, a glutamine synthetase gene (Fig 4B and 4C) and an *E*. *coli* isolate had a R276C mutation in one copy of the *dacD* gene encoding D-alanyl-D-alanine carboxypeptidase.

The number of identified duplications in this dataset is most likely an underestimate. Culturing isolates under laboratory conditions to obtain pure cultures will remove the conditions that selected the duplication and will lead to segregation unless the duplication is stabilized. The fact that multiple isolates with duplications were identified for every species shows that duplications of chromosomal regions are very common among natural isolates. These duplications were stable enough to be present after laboratory culture conditions and to acquire mutations in one of the duplicated copies. The *M*. *tuberculosis* isolate that had an inactivating frameshift mutation in one copy of a glutamine synthetase gene (Fig 4C) represents in principle the Δ1 mutant class predicted in the SNAP model (Fig 2).

## Discussion

Understanding drivers and mechanisms of genetic change is fundamental to understanding the diversity of life on earth. This diversity of lifeforms has evolved from a common ancestor by mutation and recombination of existing genetic material. Most research in this area has focused on the causes, and selection, of changes in gene sequences, and there has been much less research into the causes, and selection, of changes at the level of the chromosome [7]. Current theory interprets the widespread diversity in chromosomal gene order as evidence of very weak selection, with rearrangements occurring by genetic drift. Accordingly, rearrangements in gene order that are not counter-selected can accumulate by successive recombinational events (inversions, transpositions, deletions, and re-acquisitions by HGT) leading ultimately to a shuffled set of genes [25]. However, experimental evidence shows that most individual chromosomal rearrangements reduce fitness, creating a barrier to their fixation [28, 29]. The major advantages of the SNAP hypothesis over the genetic drift hypothesis are: (i) it is associated with an important lifestyle event (entry into a new ecological niche); (ii) it is initiated by a high-frequency event (partial chromosome duplication); (iii) it is driven by positive selection (adaptation to the new niche by increased gene dosage); (iv) selection to reduce the dosage of non-selected genes drives the loss of function or deletion of many duplicated genes; (v) the loss of essential genes in each copy of the duplicated region traps the rearrangement; (vi) a rearranged gene order becomes fixed in the niche-adapted bacterial variant. An additional consequence is that bacteria with a novel gene order will be genetically more isolated, contributing to the process of species separation in bacteria.

Most bacterial genes are organized into multigene transcriptional units, operons, that can be physiologically advantageous in terms of transcriptional co-regulation of genes with intersecting functionalities [17–19]. The organization of genes into operons is likely to act as a selective force resisting disruptive rearrangements in linear gene order within the operon if that reduces relative fitness. In this regard, finely regulated operons may be under stronger positive selection and able to resist disruptive rearrangements more than poorly regulated operons. However, even for the tryptophan operon, a classic whole-pathway operon with an ancient history (present in the common ancestor of Bacteria and Archaea), phylogenetic analysis has revealed many differences in gene order in different bacterial lineages [65]. Operons can also be advantageous for their member genes on an evolutionary timescale, by increasing the likelihood that the genes contained within the operon can benefit from horizontal gene transfer events by being transferred as part of a fully functional unit [20, 21]. Re-ordering linear gene order is however, not just a potential disrupter of operons. Rearrangements in linear gene order can act to create novel transcriptional units with potential selective value if they increase fitness of the organism [66, 67]. Accordingly, the pathway to fixation of a new gene order during the process of SNAP could involve a series of different selection processes: selection to maintain the initially selected gene dosage benefit, selection to reduce the negative effect of costly duplications, and selection to maintain fortuitously created novel regulatory units arising during the fixation process.

The SNAP hypothesis as outlined here is a dynamic process that begins with high-frequency spontaneous duplications of chromosome segments [30] that are maintained by selection for increased gene dosage [30–32, 68], and ultimately, through a process of mutation and recombination, driven by selection for high fitness, results in the fixation a new linear gene order (Fig 1B). The high frequency of chromosome segment duplications predicts that occasionally the duplication should be retained, either by selection for gene dosage or as a result of mutational fixation. Genome analyses provide evidence for some bacterial genes arising by duplication [69–74]. One interesting example is that duplicated segments have been found in the genomes of *Mycobacterial* species, ranging in size from 30 to 350 kb [75–77] suggesting that they are maintained, or very frequently generated, by selection. The frequent presence of multiple copies of ribosomal RNA operons in bacterial genomes is a classic example of duplicated genes that are stably maintained on evolutionary timescales. It is assumed that these operons have a common evolutionary origin and that the presence of multiple copies in many [37] but not all [78, 79] bacterial species is most probably explained by duplication of chromosomal regions. The selection for different copy numbers correlates closely with growth rate [37] but there is evidence that selection for adaptation to different ecological niches and for the ability to respond efficiently to the availability of resources also plays a significant role [38].

To search for genomic evidence relevant to the SNAP hypothesis we examined recent genome sequence data deposited at the Sequence Read Archive. We chose, without any pre-screening, one hundred genome sequences from each of four clinically important bacterial species: *E*. *coli*, *P*. *aeruginosa*, *A*. *baumannii*, and *M*. *tuberculosis* (SI, Table). We searched the raw sequence reads for evidence of partial chromosomal duplications (step 1 in the model), and mutations within one copy of a duplicated region (step 2 in the model). We found duplicated regions in the genome sequences of all four species at frequencies of 2 to 4%, and we also observed mutations at 50% frequency in 2 of the 14 duplicated regions (Fig 4). These mutations included one frameshift mutation in a duplicated region of *M*. *tuberculosis* that is expected to inactivate the gene (glutamine synthetase) and this represents a good example of the second step in the model (Fig 2). Given that these clinical samples do not represent bacteria encountering a novel environment, and that the genomic DNA was prepared for sequencing without special selection to maintain unstable duplications, these data show that the initial two steps in the SNAP process can occur with a remarkably high frequency.

The computational model and the genome-level analysis of natural isolates sequences indicate that the SNAP process can act on bacterial genomes. Nevertheless, so far there is no direct empirical evidence that genome rearrangements in modern bacterial species have been caused by SNAP. A complicating factor is that once the SNAP process is completed there is no genome feature left that is unique to the model. A possible bioinformatic approach to test the hypothesis would be a high-throughput analysis of modern bacterial chromosomes to search for intermediate steps of the SNAP process. For example, a larger than expected number of duplicate genes and/or pseudogenes with matching active copies could be the remains of old duplications. Alternatively, a long-term adaptation experiment of a bacterial clone to a novel environment (e.g. growth on a poor carbon source) could be analysed to experimentally identify and validate each of the proposed steps of the SNAP hypothesis.

In summary, the SNAP hypothesis is based on a sequential series of high-frequency events (ecological and genetic), that can selectively drive a process leading with a high probability to rearrangements in chromosomal gene order, and possibly also contributing to creating species barriers between bacteria.

## Methods

### Mathematical model

The mathematical model was designed to model 1000 generations of growth of a wild-type population (Wt). The model allows the appearance of cells with a small duplication (Dup) that includes two essential genes, and cells with the duplication and a single (Δ1) or double (Δ2) inactivation of essential genes. Rates of duplication formation and mutational gene inactivation were estimated based on previous experimental data [30, 46, 47]. All possible directions and rates of evolution are displayed in Fig 2 and the inactivation of two essential genes is assumed to stabilize the duplication. Fitness effects of duplications and gene inactivations were the variable parameter of the model and are displayed in Fig 3.

The bacterial growth rate is a monotonically increasing function of the concentration of a limiting resource, R (mg L^-1^) [80]

$$\psi_{i}(R)=V_{i}\left(\frac{R}{R+k}\right)$$ (1)

where V_i_ is the relative fitness of the ith strain of bacteria and k is the concentration of the resource at which V_i_ is at half its maximum value. With these definitions the change in densities of bacterial populations and the concentration of resources are given by the following two coupled differential equations:

$$\frac{dR}{dt}=-\sum_{i=1}^{2}n_{i}*\psi_{i}(R)*e$$ (2)

$$\frac{dn_{i}}{dt}=n_{i}*\psi_{i}(R)$$ (3)

where n_i_ is the density of strain i (cfu mL^-1^) and e is the conversion efficiency parameter (μg cell^-1^). The standard parameters R_t = 0_ = 100 mg L^-1^, k = 1 mg L^-1^, and e = 10^−9^ μg cell^-1^ result in a growth cycle that leads to a final density of approximately 10^10^ cfu mL^-1^. After every cycle the culture is 100-fold diluted (10^8^ cells per bottleneck) into fresh media and grown to full density. Serial passaging was repeated until a total growth of 1000 generations. A Monte Carlo procedure was used to determine the appearance of Wt, Dup, Δ1 and Δ2 cells. The probability p_i>j_(t) that a cell j is generated from a cell i at time point t is

$$p_{i>j}(t)=g_{i}*\mu_{i>j}$$ (4)

where g_i_ is the number of generations of growth of the strain i at time point t, and μ_i>j_ is the mutation/recombination rate to generate cell j from cell i. A random number x (0 < x < 1) is generated. A single cell of strain j generated at time point t if x < p_i>j_(t). The simulation was programmed in Berkeley Madonna (Version 9.1.14) and run with varying fitness values. All results are averages of 100 independent simulations.

### Analysis of natural isolates

Genome analyses were performed using the CLC Genomics Workbench version 11.0.1 (Qiagen). Whole genome sequencing reads were downloaded from the Sequence Read Archive for one hundred natural isolates per species (S1 Table). The downloaded reads were trimmed and mapped to a respective standard reference sequence (Trim settings: Quality limit: 0.05; Ambiguous limit: 2. Mapping settings: Match score: 1; Mismatch score: 2; Cost of insertions and deletions: Linear gap cost; Insertion cost: 3; Deletion cost: 3; Insertion open cost: 6; Insertion extend cost: 1; Deletion open cost: 6; Deletion extend cost: 1; Length fraction: 0.5; Similarity fraction 0.8; Auto-detect paired distances; Non-specific match handling: Map randomly. Reference sequences: *A*. *baumannii* str. ACICU: NC_010611; *E*. *coli* K-12 str. MG1655: NC_000913; *M*. *tuberculosis* str. H37Ra: NC_009525; *P*. *aeruginosa* str. PAO1: NC_002516). Duplications were identified based on visual assessment of the CLC sequence coverage tracks. See Fig 4B for an example of an identified duplication. All isolates containing duplications are highlighted yellow in S1 Table.

## Supporting information

S1 Table SRA metadata tables. SRA metadata for all isolates included in the study. Isolates with duplications are highlighted in yellow.
(XLSX)
